# Supplementary material for: Cirsilineol improves anesthesia/surgery-induced postoperative cognitive dysfunction through attenuating oxidative stress and modulating microglia M1/M2 polarization
Source: PeerJ. 2024 Nov 15;12:e18507. doi: 10.7717/peerj.18507 (PMC11572359; doi:10.7717/peerj.18507)
Supplement: Supplemental Information 3 [file peerj-12-18507-s003.zip › WB for Original blot.pptx]

## Slide 1
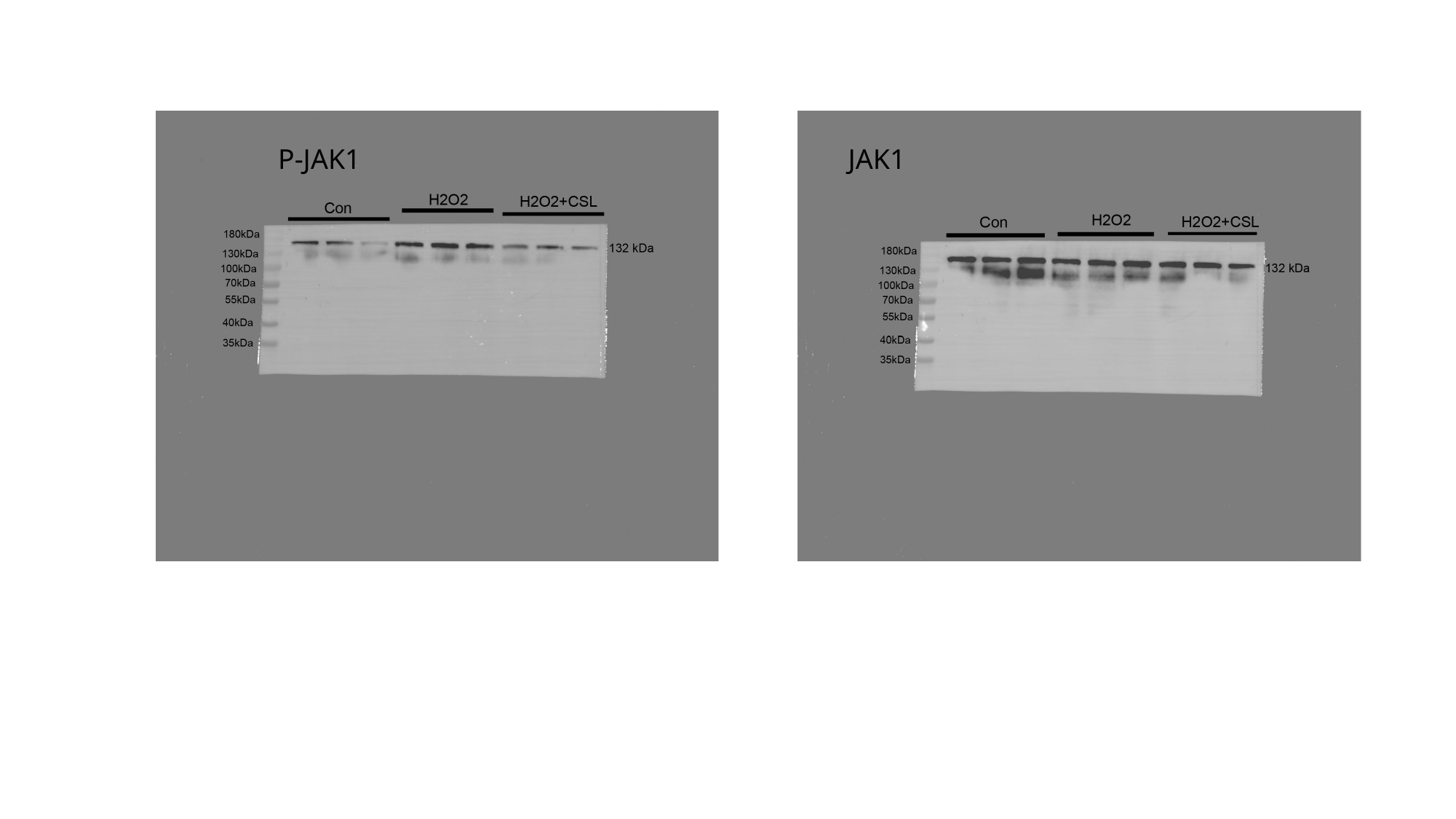

P-JAK1
JAK1

## Slide 2
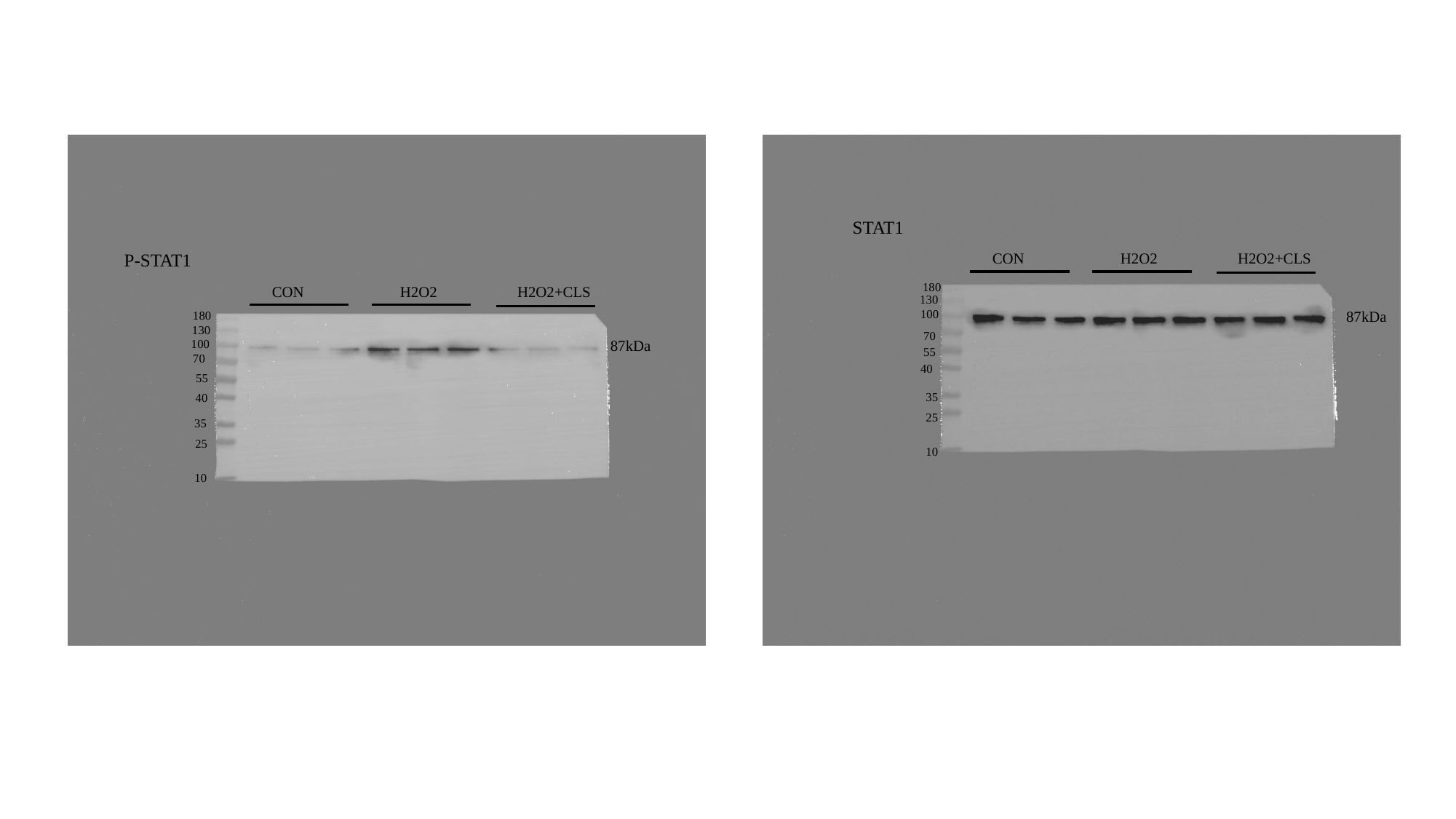

STAT1
P-STAT1
CON
H2O2
H2O2+CLS
180
CON
H2O2
H2O2+CLS
130
100
87kDa
180
130
70
100
87kDa
55
70
40
55
35
40
25
35
25
10
10

## Slide 3
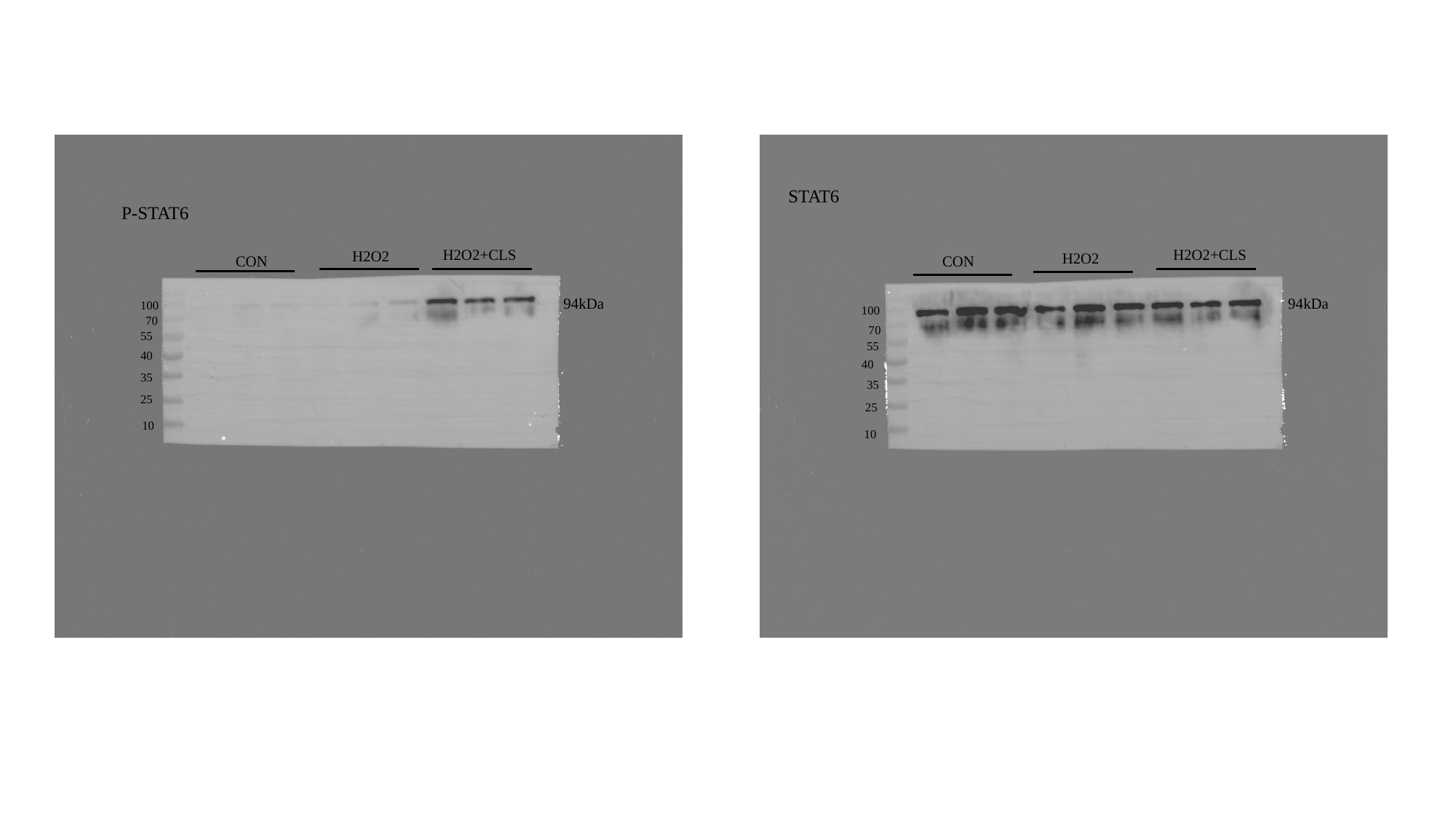

STAT6
P-STAT6
H2O2+CLS
H2O2+CLS
H2O2
H2O2
CON
CON
94kDa
94kDa
100
100
70
70
55
55
40
40
35
35
25
25
10
10
